# Risk assessment of oxidative stress and multiple toxicity induced by Etoxazole

**DOI:** 10.1038/s41598-022-24966-0

**Published:** 2022-11-28

**Authors:** Oksal Macar, Tuğçe Kalefetoğlu Macar, Kültiğin Çavuşoğlu, Emine Yalçın

**Affiliations:** 1 Department of Food Technology, Şebinkarahisar School of Applied Sciences, Giresun University, Giresun, Turkey; 2 Department of Biology, Faculty of Science and Art, Giresun University, Giresun, Turkey

**Keywords:** Cytogenetics, Enzymes, Chromosomes

## Abstract

Etoxazole is among the systemic pesticides with acaricidal and insecticidal characteristics. This paper reports the first evaluation of the toxic effects of Etoxazole on *Allium cepa* L. Etoxazole solutions were applied to three groups formed from *A. cepa* bulbs at 0.125 mL/L, 0.25 mL/L and 0.5 mL/L doses, respectively. The control group was treated with tap water throughout the experimental period. The toxic effects of Etoxazole became more apparent as the dose of Etoxazole was increased. The growth-limiting effect was most pronounced in the highest dose group with approximately 29%, 70% and 58.5% reductions in germination percentage, root elongation and weight gain, respectively. The genotoxic effect of Etoxazole was most severe in the 0.5 mL/L dose group. In this group, the mitotic index decreased by 30% compared to the control group, while the micronucleus frequency increased to 45.3 ± 3.74. The most observed aberrations were fragment, vagrant chromosome, sticky chromosome, unequal distribution of chromatin, bridge, reverse polarization and nucleus with vacuoles. The malondialdehyde level showed a gradual increase with increasing Etoxazole doses and reached 2.7 times that of the control group in the 0.5 mL/L Etoxazole applied group. Catalase and Superoxide dismutase activities increased in the groups exposed to 0.125 mL/L and 0.25 mL/L Etoxazole with dose dependence and decreased abruptly in the group treated with 0.5 mL/L Etoxazole. Etoxazole triggered meristematic cell damages, such as epidermis cell damage, thickening of cortex cell walls, flattened cell nucleus and indistinct transmission tissue. Considering the versatile toxicity induced by Etoxazole, we announce that this chemical has the potential to cause serious damage to non-target organisms. It should be noted that the higher the dose of exposure, the more severe the level of damage. This study will be an important reminder to limit the indiscriminate use of this highly risky agrochemical.

## Introduction

The main threats to agriculture and forestry around the world are pest-related plant diseases^1^. Pesticides are chemical and natural substances administered to control or kill pests, including insects, herbs, rodents, arthropods and nematodes that cause plant diseases, yield losses and health problems^2^. Due to the widespread use of pesticides in many agricultural industries, high levels of agricultural production and quality have been achieved. In addition to nourishing and multiplying crop yield, pesticides have other benefits, such as saving time and effort^3^. Despite all their advantages, pesticides, most of which are hydrophilic, tend to accumulate in nature as potential pollutants and harm non-target organisms^4^. Common classifications of pesticides are based on the target pest.

Pesticides used to combat mite populations include chemicals with acaricidal/insecticidal properties. Etoxazole, a diphenyl oxazoline miticide, is chemically known as 2-(2,6-difluorophenyl)-4-[4-(1,1-dimethylethyl)-2-thoxyphenyl]-4,5-dihydrooxazole^5^. It was first introduced to the public as a new option to carbamates, pyrethroids, organophosphates and organochlorines in 1994 and took its place in the market as an acaricide/insecticide in 1998^6^. Since that time, it has been utilized to eliminate the eggs, larvae and nymphs of mites including *Tetranychus* spp., *Eotetranychus* spp. and *Panonychus* spp. on fruits such as grapes, strawberries, nuts, citrus and cotton^5,7^. Pest management achieved by Etoxazole also includes limiting damage from rice green leafhopper (*Nephotettix nicropictus*) and diamondback moth (*Plutella xylostella*) infestations^8^. Etoxazole inhibits chitin biosynthesis by binding to the sulfonylurea receptor and prevents moulting^9^. Chang et al.^5^ investigated the systemic stereoselectivity of Etoxazole in grapes, strawberries and apples to estimate residue-induced toxicity in humans and the environment. Due to its 20-day half-life, Etoxazole residues can be easily transported to humans and animals. According to EFSA^10^, Etoxazole can be accepted as bio-accumulative, persistent and toxic. It has been reported that Etoxazole induces toxic effects such as neurotoxicity, genotoxicity, cytotoxicity, infertility, endocrine disrupting activity and oxidative stress on various non-target organisms^9,11–16^. In zebrafish (*Danio rerio*) larvae, Park et al.^4^ discovered that Etoxazole caused developmental abnormalities, hearth rate disorders and reduced viability. In the study of Ham et al.^9^, it was indicated that Etoxazole exposure induced testicular toxicity arisen from mitochondrial failures and altered gene expression in mice. Etoxazole-induced genotoxicity in human peripheral lymphocytes was evidenced by an increase in chromosome abnormalities and a decrease in mitotic division^11^. The European Union banned the use of Etoxazole in 2018 because of its persistence in soil sediments and toxicity^17^.

The overuse of pesticides raises concerns about their safety. There are many eukaryotic bioassay methods used to evaluate pesticide-induced toxicity in non-target biota. With its large chromosomes (2n = 16) and rapid root growth, the *Allium cepa* test system is well suited for determining the cytotoxicity and genotoxicity of pesticides. As a short-term indicator, it allows assessing the score of the mitotic index (MI), the frequencies of micronuclei (MN) and aberrant chromosomes (ACs), as well as the levels of oxidative stress and growth^18^. The most important merit of this test system is that the results obtained from the *A. cepa* assay have an extremely high correlation with analyses in mammals^19^.

In the literature, there is a data gap in the field reporting the multifaceted toxic effects of Etoxazole applications on plants, apart from residue analyses. Therefore, the object of the present study was to determine the biological responses of *A. cepa* to Etoxazole. For this purpose, physiological (rooting percentage, root elongation and weight increase), genotoxic (MI, MN and ACs) and biochemical catalase (CAT: EC 1.11.1.6) and (superoxide dismutase (SOD: EC 1.15.1.1) activities and malondialdehyde (MDA) accumulation effects in Etoxazole-treated *A. cepa* were evaluated. Moreover, the types of cellular damage induced by Etoxazole in meristematic tissue were analyzed.

## Materials and methods

### Preparation of test materials

**Figure 1:**
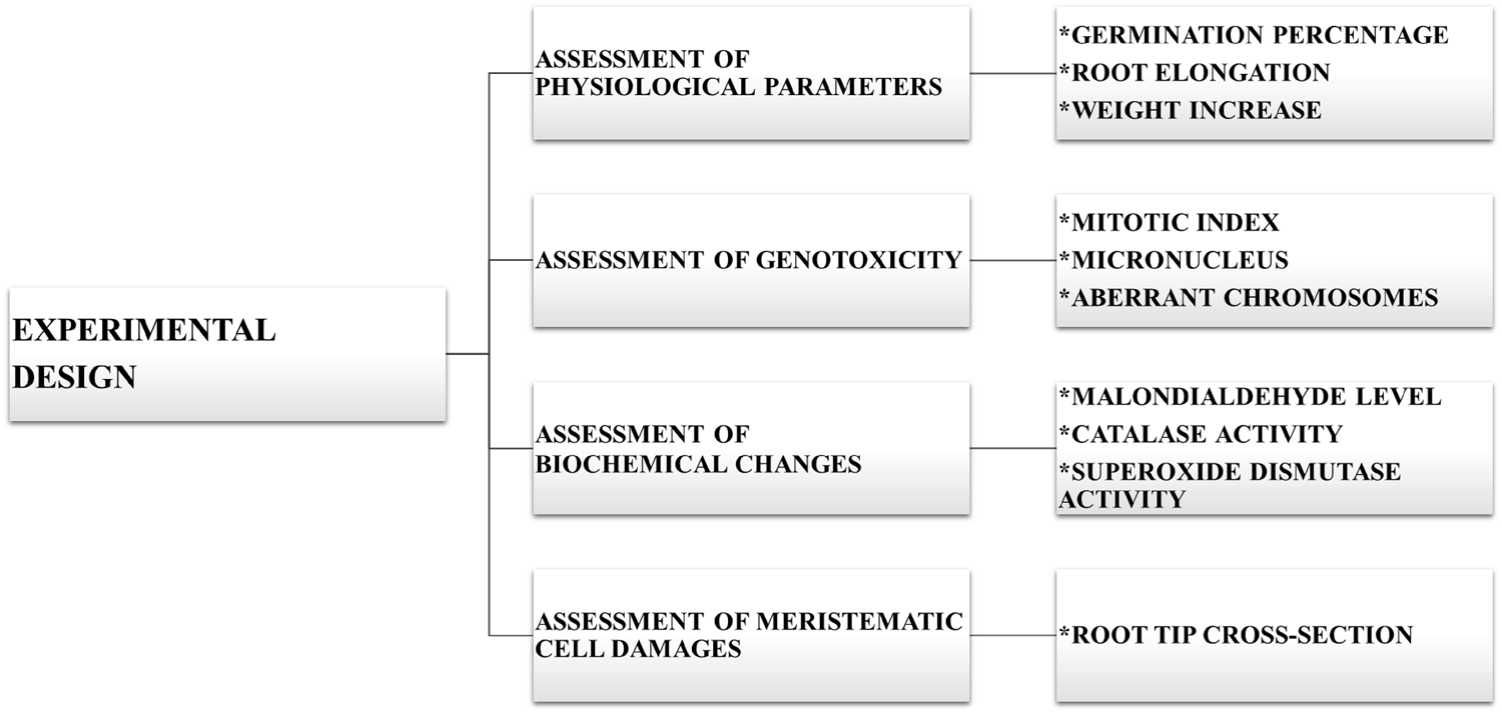
Flow diagram of experimental design.

*A. cepa* bulbs obtained commercially from Şebinkarahisar district of Giresun province were classified in the laboratory based on their size. The bulbs with the closest initial weights to each other were selected for the study. Four groups (each containing 50 bulbs) were formed. Aqueous solutions of Etoxazole were prepared from the commercial preparation with the trade name Delos (Hektaş Ticaret Türk A.Ş., Kocaeli, Türkiye) containing 110 g/L Etoxazole as the active ingredient. Etoxazole solutions were applied to the three treatment groups at doses of 0.125 mL/L, 0.25 mL/L, and 0.5 mL/L, respectively. In the selection of Etoxazole concentrations, the application dose recommended by the manufacturer, and half and twice this dose were used. On the other hand, the control group was treated with tap water during the experiment. All treatments were carried out by placing the onion stems in glass tubes in contact with the relevant solution to allow the emergence of fresh adventitious roots. The solutions were regularly refreshed every day. The application of pesticide solutions was carried out in a dark room at a constant temperature (23 ± 2 °C) for 3 days. In order to investigate Etoxazole toxicity, multifaceted analyses were performed in *A. cepa* (Fig. 1).

The use of plants and the experiments in the present study complies with the relevant international, national and institutional guidelines.

### Assessment of physiological parameters

In the determination of germination percentage, bulbs with roots longer than 1 cm were estimated as “rooted”. While calculating the germination percentage, the total number of rooted bulbs was divided by the total number of bulbs and the result was given as a percentage (%). To observe the rooting percentage, 50 bulbs from each group (n = 50) were taken into account.

All bulbs were weighed (g) before administration of the relevant solutions and after 72 h of treatment. The weight recorded before the applications was subtracted from the second weight to calculate the total weight increase of the bulbs.

At the end of the experiment, the length of the roots (cm) from the apex of the root cap to the root primordial was measured with a ruler to evaluate root elongation. For root elongation and weight gain, 10 randomly selected bulbs from each group (n = 10) were analyzed.

### Assessment of genotoxicity

Genotoxicity measurements, including MI, MN and ACs analyses, were carried out by microscopic examination of decapitated root tip preparations^20^. Root tips were placed in Clarke’s fixator consisting of glacial acetic acid and ethanol (3:1) for 120 min following the harvest. Root materials were then hydrolyzed in a 1 N HCl solution at 60 °C for 12 min. A 1% acetocarmine solution freshly prepared in 45% acetic acid was utilized to stain root tips for 24 h. Microscopic preparations were formed by gently crushing root materials with a drop of 45% acetic acid under a coverslip. For genotoxicity analyses, 10 slides were prepared from each group. MI analysis was performed by screening 10,000 randomly selected cells from these slides (1000 cells per slide). On the other hand, MN and ACs were evaluated by screening 1000 randomly selected cells from the same slides (100 cells per slide). MN existence was evaluated using the features previously noted by Fenech et al.^21^. All slides were screened under a research microscope (Irmeco, IM-450 TI) with a magnification of X 500.

### Assessment of biochemical changes

In order to determine the biochemical changes caused by Etoxazole applications, the MDA level and the activities of CAT and SOD enzymes were investigated.

The extraction of antioxidant enzymes was performed according to Zou et al.^22^. 0.1 g of root tip material was mixed with 1 ml of sodium phosphate buffer after it was thoroughly ground by adding liquid nitrogen to it. The pH of the buffer was adjusted to 7.8 before the buffer (50 mM) was used. The homogenates were immediately centrifuged at 14,000 rpm at 4 °C for 20 min, and the upper fraction (the supernatant containing the enzyme) was stored in a deep freezer at − 80 °C.

The catalytic activity of the CAT enzyme was assessed according to the analysis method of Beers and Sizer^23^. Firstly, 1.5 mL of sodium phosphate buffer at 0.2 M concentration and 7.8 pH was taken into a tube. The buffer was mixed with hydrogen peroxide (0.1 M) and distilled water. The reaction was started by adding the enzyme-containing buffer (0.2 mL) to the mixture. CAT activity was calculated as OD_240 nm_ minute per g of fresh weight (OD_240 nm_ min/g FW) by monitoring the absorbance, showing the decrease in hydrogen peroxide concentration at 240 nm wavelength. The whole procedure was repeated 10 times (n = 10).

The catalytic activity of the SOD enzyme was assessed according to the analysis method of Beauchamp and Fridovich^24^. Firstly, 1.5 mL of sodium phosphate buffer at 0.05 M concentration and 7.8 pH was taken into a tube. Distilled water, EDTA-Na_2_ (0.1 mM), riboflavin (20 μM), methionine (130 mM), nitro blue tetrazolium chloride (750 μM) and polyvinylpyrrolidone (4%) were added to the buffer-containing tube. Finally, 0.01 mL of enzyme-containing buffer, which was dissolved by holding the tubes in the palm of the hand without shaking, was added to this mixture. For the initiation of enzyme activity, the mixtures were exposed to fluorescent light of 375 μmol/m^2^/s intensity for 15 min. SOD activity was calculated as a unit per mg of fresh weight (U/mg FW) by reading the absorbance of the samples at 560 nm wavelength. The whole procedure was repeated 10 times (n = 10).

MDA accumulation, which is a common marker of peroxidation of the lipids in cellular membranes, was analyzed according to Unyayar et al.^25^. 0.4 g of root tip material was ground in a mortar containing 8 mL of trichloroacetic acid (5%). Following the homogenization completed at room temperature, the samples were centrifuged at 12,000 rpm for 15 min in a centrifuge at 23 °C. The upper fraction of the homogenates was collected and added to the premixed trichloroacetic acid (20%) and thiobarbituric acid (0.5%). After allowing the reaction to proceed at 90 °C for 40 min, the reaction was stopped by placing the tubes on ice. A second centrifugation process (10,000 rpm for 5 min) was applied to obtain the supernatant fraction. The MDA levels of the samples were calculated as μM per g of fresh weight (µM/g FW) by recording the absorbance of the supernatant fractions at 532 nm wavelength. The whole procedure for MDA determination was repeated 10 times (n = 10).

### Assessment of meristematic cell damages

Meristematic cell damages triggered by Etoxazole applications were assessed in the cross-section of the roots taken from the root tips, excluding the cap part. Roots were carefully washed to remove pesticide residues before sections were prepared. Two drops of methylene blue (3%) were used to stain the cells. In order to analyze meristematic abnormalities, slides were screened under a research microscope (Irmeco, IM-450 TI) with × 500 magnification. Three severity classes were used to express the frequency of occurrence of damage types: absent, minor, moderate and major.

### Statistical analysis

One-way ANOVA and Duncan’s test were implemented to determine the statistical significance (*p* < 0.05) between the mean values. Results were arranged to specify “mean value + standard deviation”.

## Results and discussion

Table 1 shows the “growth arrest” induced by Etoxazole treatments. While the deleterious effect of Etoxazole on physiological parameters was most pronounced in the HLE group, the least inhibition was observed in the LLE group. The high germination percentage determined in the control group was proof that the bulbs selected for the experiment were healthy. The final root length and the mean weight increase of the control group were 12.0 ± 0.84 cm and 8.09 g, respectively. There was a significant reduction in root elongation and weight increase compared to the control, even in LLE, the group that received the lowest dose of Etoxazole. Indeed, root elongation and weight increase values in HLE were decreased by 70% and 58%, respectively, when compared to the control group values. The dose-dependent effects of various pesticides on the growth and development of non-target organisms have been demonstrated in different investigations before^26–29^. Although the effect of Etoxazole on growth in plants has not been previously investigated, it has been shown that growth is suppressed in zebrafish embryos exposed to this pesticide^4^. Park et al.^4^ demonstrated that the growth-limiting mechanism is due to the fact that Etoxazole both suppresses mitotic division and induces programmed cell death. Amaç and Liman^27^ suggested that genotoxicity and excessive radical production may be the reason why agrochemicals inhibit cell division in onion root cells. In this study, the inhibitory power of Etoxazole on growth-related physiological parameters was demonstrated for the first time in *A. cepa* roots.

**Table 1:** Etoxazole toxicity on selected physiological parameters.

Groups	Germination Percentage (*%*)	Root Elongation (*cm*)	Weight Increase (*g*)
Control	99	12.0 ± 0.84^a^	+ 8.09^a^ (12.05 ± 1.28–20.14 ± 1.55)
LLE	88	9.80 ± 0.44^b^	+ 6.58^b^ (11.83 ± 1.15–18.41 ± 1.32)
MLE	81	7.20 ± 0.51^c^	+ 4.64^c^ (11.55 ± 1.41–16.19 ± 1.54)
HLE	70	3.60 ± 0.29^d^	+ 3.36^d^ (11.51 ± 1.32–14.87 ± 1.31)

*Control: Tap water, *LLE* Low level Etoxazole (0.125 mL/L), *MLE* Medium level Etoxazole (0.25 mL/L), *HLE* High level Etoxazole (0.5 mL/L). The superscript letters ^(a–d)^ in the same column indicate the statistical significance between the mean values (*p* < 0.05).

**Figure 2:**
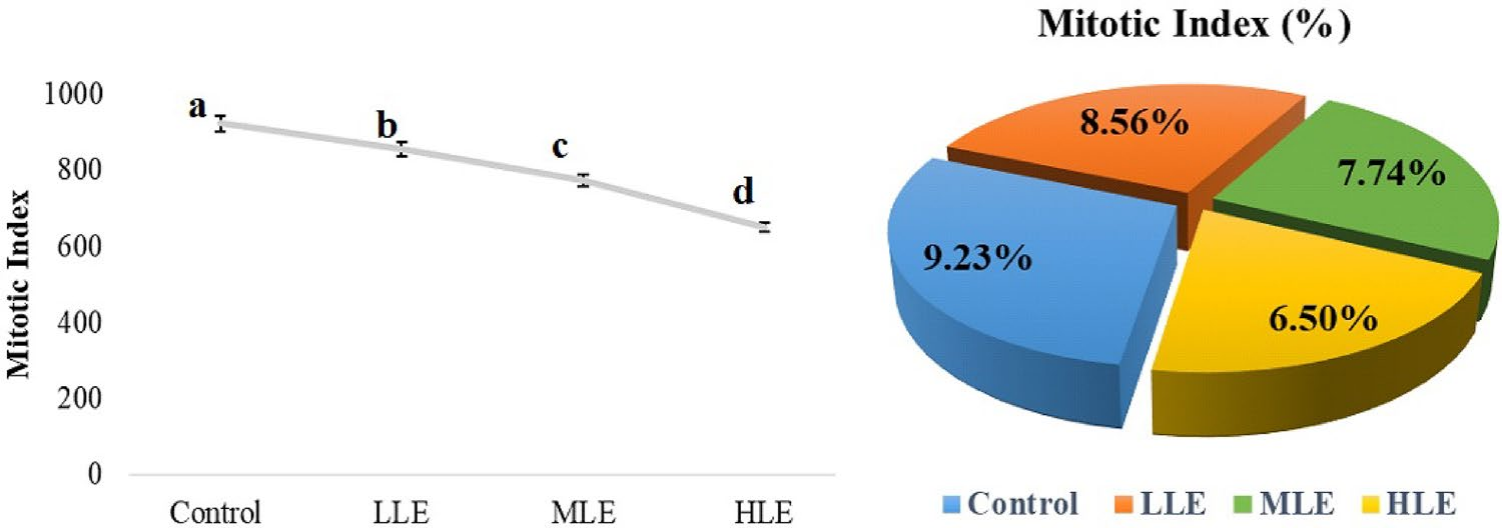
The effect of Etoxazole on MI and MI (%). Control: Tap water, *LLE* Low level Etoxazole (0.125 mL/L), *MLE* Medium level Etoxazole (0.25 mL/L), *HLE* High level Etoxazole (0.5 mL/L). Different letters (a–d) indicate the statistical significance between the mean values (*p* < 0.05).

The reduction in MI values in all Etoxazole exposed groups was concomitant with growth inhibition (Fig. 2). The MI scores of the bulbs in the LLE, MLE and HLE groups declined gradually. The differences between the MI results of all groups were statistically significant. The MI was defined as an easily reproducible, specific, and highly sensitive biomarker of cytostaticity and cytotoxicity^30^. The suppressive activity of Etoxazole in MI levels of *A. cepa* was certainly dose-related. Gogoi et al.^31^ noted that a gradual decrease in MI is a hallmark of the toxicity of any contaminant upon mitotic division of cells. According to Shabbir et al.^32^, MI values lower than the control value point out that the pollutants to which the cells are exposed interfere with the growth and development of organisms by limiting cell division. Although this study showed for the first time that Etoxazole slows down the division of *A. cepa* root tip cells, the mitodepressive effect associated with the other pesticide types, which can be predicted by MI analysis, has been shown in *A. cepa* roots by many researchers before^33–35^. In addition, Rencüzoğullari et al.^11^ reported that increasing Etoxazole concentrations triggered a significant decrease in the MI value of human lymphocytes, depending on the application dose. The microtubules, which form the spindle apparatus in the cell, ensure the proper separation of sister chromatids during cell division. Pesticides that interact with tubulins to interrupt microtubule polymerization may prevent chromosome movement towards the poles, triggering both the disruption of mitotic order and the formation of MN and ACs^36^.

**Figure 3:**
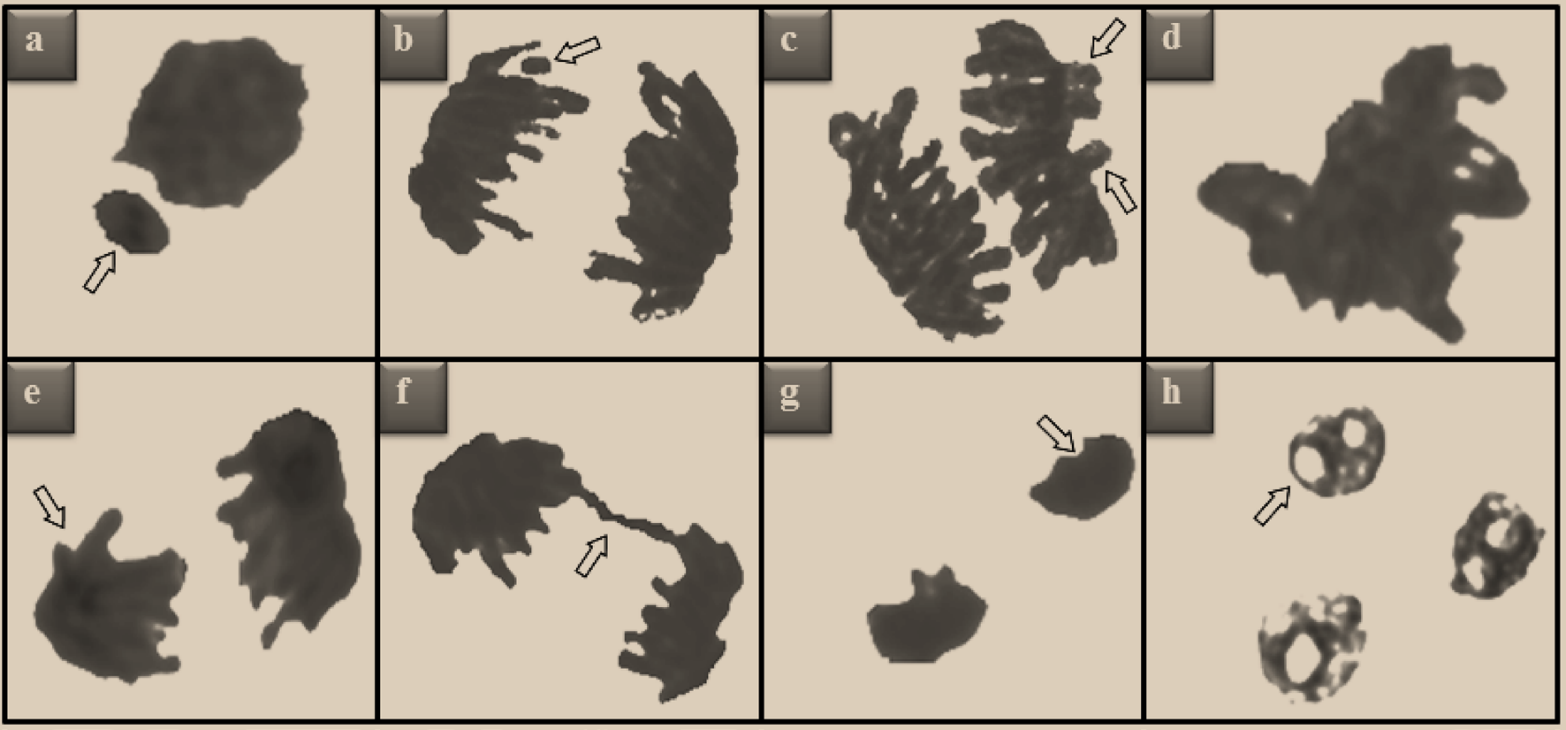
AC types induced by Etoxazole administration. MN (**a**), fragment (**b**), vagrant chromosome (**c**), sticky chromosome (**d**), unequal distribution of chromatin (**e**), bridge (**f**), reverse polarization (**g**), nucleus with vacuoles (**h**).

The quantification of MN often serves as an explicit index of genotoxicity as well as chromosomal instability. In addition, MN analysis provides information on genetic material defects, defects in mitosis and responses to stress factors^37^. A remarkable increment in MN formation was observed in all groups exposed to Etoxazole (Table 2). However, the most prominent MN (Fig. 3a) frequency was determined in the HLE group. MN seems like a miniature version of the cell nucleus and appears as a result of lagging chromosomes or acentric chromosomes encapsulated in an abnormal nuclear envelope^38^. Rencüzoğullari et al.^11^ pointed out that the formation of MN and ACs following Etoxazole administration may be due to the breaking of the phosphodiester skeleton in DNA. In addition, some researchers noted that pesticides generally cause DNA ruptures as well as kinetochore, centromere and spindle impairments to generate MN^39,40^. The present study is the first in the literature to reveal the MN-inducing capacity of Etoxazole in plants.

**Table 2:** Genotoxicity induced by Etoxazole administration.

Damage types	Control	LLE	MLE	HLE
MN	0.14 ± 0.33^d^	14.4 ± 1.13^c^	26.8 ± 1.97^b^	45.3 ± 3.74^a^
FRM	0.00 ± 0.00^d^	12.6 ± 1.10^c^	24.7 ± 1.84^b^	41.9 ± 3.52^a^
VGC	0.00 ± 0.00^d^	10.7 ± 0.96^c^	21.6 ± 1.65^b^	37.1 ± 3.16^a^
STC	0.12 ± 0.28^d^	8.1 ± 0.88^c^	18.7 ± 1.51^b^	33.2 ± 2.74^a^
UDC	0.10 ± 0.24^d^	6.5 ± 0.79^c^	15.5 ± 1.28^b^	28.6 ± 2.62^a^
BRG	0.00 ± 0.00^d^	5.4 ± 0.65^c^	13.4 ± 1.15^b^	24.7 ± 2.33^a^
RVP	0.00 ± 0.00^d^	4.1 ± 0.58^c^	9.2 ± 0.94^b^	16.8 ± 1.48^a^
NV	0.00 ± 0.00^d^	2.8 ± 0.44^c^	6.3 ± 0.69^b^	13.7 ± 1.20^a^

*Control: Tap water, *LLE* Low level Etoxazole (0.125 mL/L), *MLE* Medium level Etoxazole (0.25 mL/L), *HLE* High level Etoxazole (0.5 mL/L). The superscript letters (a–d) in the same line indicate the statistical significance between the mean values (*p* < 0.05). *MN* micronucleus, *FRM* fragment, *VGC* vagrant chromosome, *STC* sticky chromosome, *UDC* unequal distribution of chromatin, *BRG* bridge, *RVP* reverse polarization, *NV* nucleus with vacuoles.

Cell proliferation dysregulation upon Etoxazole administration occurred simultaneously with the emergence of various types of ACs (Table 2 and Fig. 3). Etoxazole administration in the LLE, MLE and HLE groups engendered a variety of ACs in a dose-related manner (Table 2). The most seen AC types in all treatment groups were listed as fragment (Fig. 3b), vagrant chromosome (Fig. 3c), sticky chromosome (Fig. 3d), unequal distribution of chromatin (Fig. 3e), bridge (Fig. 3f), reverse polarization (Fig. 3g) and vacuole nucleus (Fig. 3h), respectively, considering their frequencies. Based on the results of many epidemiological, in vitro or in vivo studies, it can be deduced that several pesticides cause toxicity in the genomic construct^41^. Moreover, intrinsic circumstances such as inhibited or altered DNA replication and DNA damage may induce chromosomal perturbations upon exposure to various contaminants^32^. In the current study, the detrimental effect of Etoxazole on the arrangement and integrity of chromosomes in *A. cepa* roots was revealed for the first time. However, the hazards of Etoxazole application on the chromosomal regularity of human lymphocytes have been introduced earlier as well^11^. Since they are directly related to MN formation^42^, it was not surprising that fragment (Fig. 3b) and vagrant (Fig. 3c) were the most common ACs in the Etoxazole groups. Indeed, the number of fragments and vagrants in the etoxazole treated HLE group was approximately 40% of the total ACs. Fragment formation indicates the clastogenic effect of a chemical, which leads to a partial loss of genetic material^43^. Vagrant chromosomes result from spindle defects and eventually lead to the formation of irregularly shaped or unevenly sized nuclei in daughter cells^44^. According to Adrovic et al.^45^, stickiness (Fig. 3d), the third most frequent ACs in our study, is induced by toxicity and inevitably results in cell death. In fact, the main reason for the increase in chromosomal stickiness is the disorders in the nucleic acid metabolism of the cell^46^. Unequal distribution of chromatin (Fig. 3e), one of the most abundant ACs induced by Etoxazole, is such an anomaly that it is a consequence of unseparated chromatins and is accountable for the increase in vagrants^47^. On the other hand, sticky chromosomes leading to failure of separation at the anaphase stage are cited as the probable cause of bridge (Fig. 3f) formation, which is an indicator of a complete disruption of the chromosome structure^46^. Demirtaş et al.^48^ stated that both the unequal distribution of chromatin and reverse polarization (Fig. 3g) are produced due to the aneugenic effects of pollutants. The nucleus with vacuoles (Fig. 3h), the least frequent ACs in our study, points out the suppression of DNA biosynthesis because of a nuclear poison^49^. The increase in MN and ACs frequencies together with the decrease in MI value clearly shows that Etoxazole acaricide caused serious genotoxicity in *A. cepa*. Our results are in agreement with the previous findings of Kalefetoğlu Macar^50^, who showed that Abamectin, another acaricide, causes various ACs such as MN, sticky chromosome, bridge, fragment, unequal distribution of chromatin and nuclear abnormality in *A. cepa* root cells. In another study, Spirodiclofen, a widely used insecticidal agent, was proven to cause MN, fragment, bridge, sticky chromosome, vagrant, unequally distributed chromatins, budded nucleus and spindle thread disorder^51^. Although toxic effects of Etoxazole on different organisms and cell cultures have been reported previously, the mechanism underlying its toxicity in non-target organisms is still unknown^4,11,16,17^. However, Park et al.^4^ demonstrated that Etoxazole exposure promoted cell cycle inhibition and the formation of reactive oxygen species. Therefore, the likely cause of Etoxazole-induced genotoxicity is the direct interaction of these molecules with chromosomes or oxidative imbalance due to radical formation in cells.

**Figure 4:**
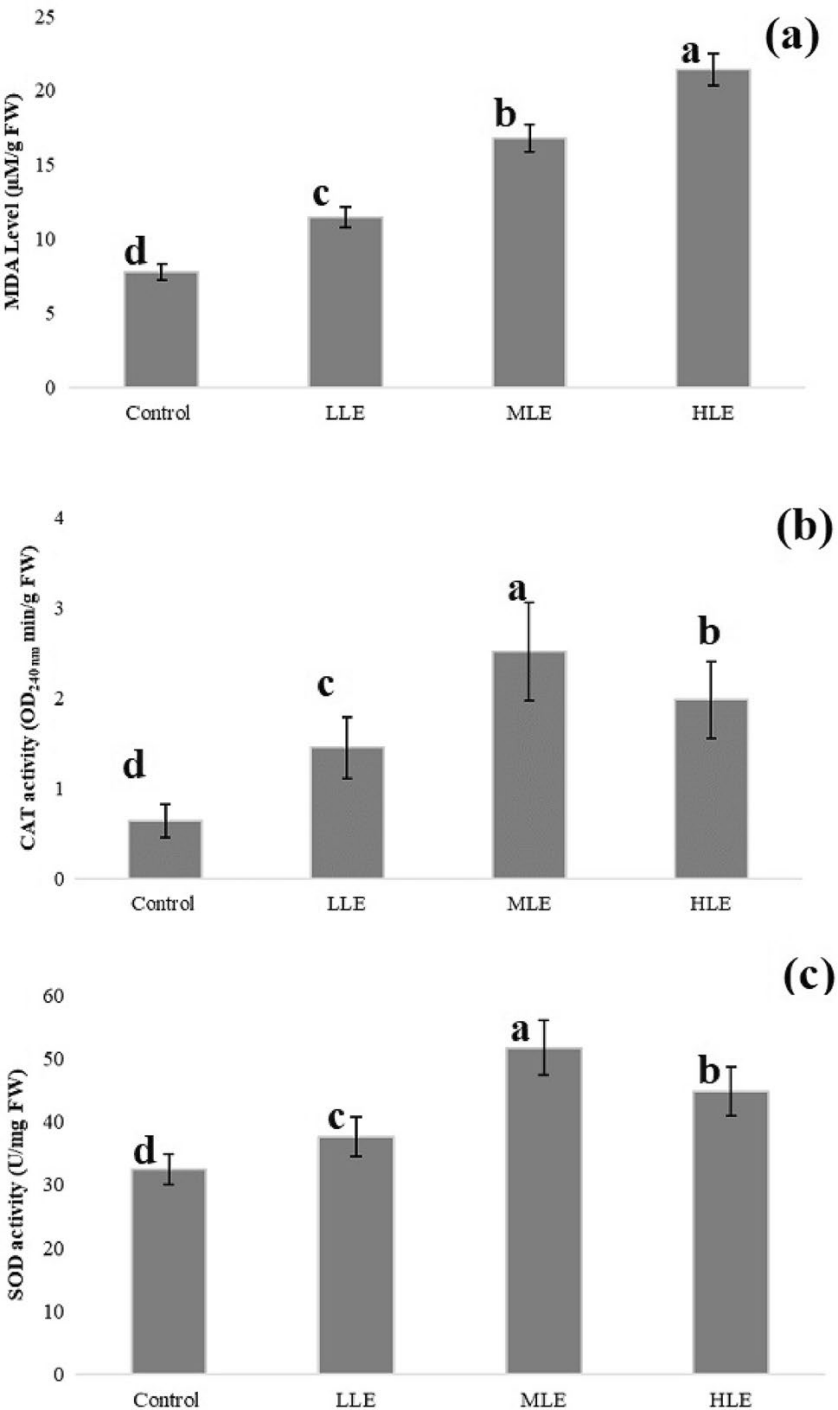
Etoxazole toxicity on selected biochemical parameters. Control: Tap water, LLE: Low level Etoxazole (0.125 mL/L), MLE: Medium level Etoxazole (0.25 mL/L), HLE: High level Etoxazole (0.5 mL/L).

CAT and SOD activities and the amount of MDA were analyzed in order to understand whether the growth retardation and genotoxicity that occurred following Etoxazole administration were related to oxidative stress (Fig. 4). As a broadly utilized indicator of membrane lipid peroxidation, the MDA level provides an estimate of oxidative stress damage in cells^52^. Etoxazole treatment at all doses triggered a remarkable rise in MDA levels compared to the control group (Fig. 4a). In addition, the differences between the MDA results of all groups were statistically significant. The MDA level in the HLE group was approximately 2.7 times that of the control group. The gradual increase in MDA accumulation in *A. cepa* root cells evidenced that a dose-related oxidative damage occurs following Etoxazole applications. Küçükakyüz et al.^53^ suggested that pesticide-associated MDA accumulation shows cell membrane rupture in tomatoes. Although there is no publication on the oxidative stress induced by the presence of Etoxazole in plants, it has been reported that the MDA level ascends as the exposure time to high dose Etoxazole increases in experimental animals^16,17^.

Abnormal circumstances may give rise to the over-production of superoxide anion and hydrogen peroxide^15^. As a member of the “antioxidant team” within the cells, the SOD enzyme catalyzes the depletion of the superoxide radical in order to reverse oxidative burst^54^. On the other hand, CAT has an extremely high turnover number and plays a crucial role in swiftly converting hydrogen peroxide into water and oxygen^55^. Etoxazole applied at different doses caused significant changes in CAT and SOD activities (Fig. 4b and c). CAT and SOD activities in the LLE group were approximately 2.3 and 1.2 times greater than those of their controls, respectively. In the MLE group, CAT and SOD activities were nearly 1.7 and 1.4 times greater than those of the LLE groups, respectively. These results obtained from the application of relatively lower doses of Etoxazole were in agreement with the study of Sun et al.^15^, which showed Etoxazole-related increases in CAT and SOD activities in cell culture. Additionally, Chang et al.^17^ reported a significant increment in SOD activity in experimental animals upon Etoxazole administration. However, different results have been reported in the literature on antioxidant enzyme behaviors after Etoxazole exposure. For instance, in the study of Yilmaz et al.^16^, CAT activity decreased in the liver and kidneys of rats exposed to different doses of Etoxazole. In our study, the tendency to increase in the catalytic activities of both enzymes was reversed in the HLE group. Although SOD and CAT activities in the HLE group were significantly higher than those in both the control and LLE groups, they were significantly lower than the values determined in the MLE group. The increase in both MDA and antioxidant enzyme activities in the first two doses of Etoxazole (in the LLE and MLE groups) was a sign that the membranes were damaged due to oxidative stress, although the defense mechanism against this stress was activated. While MDA continued to accumulate in the HLE group, enzyme activities began to be restricted; this may indicate that the enzymatic antioxidant defense mechanism of cells is no longer able to overcome oxidative stress conditions. This is the first study to demonstrate Etoxazole-induced oxidative stress in plants. Synchronized increase of oxidative stress and genotoxic damage in Etoxazole applied *A. cepa* revealed an “Etoxazole-mediated genotoxicity—oxidative stress relationship”. As a matter of fact, the increased production of reactive molecules in plant cells under the influence of agrochemicals, including pesticides, causes genotoxicity as well as oxidative stress as it damages vital elements in cells such as proteins, membranes and nucleic acids^56^.

**Figure 5:**
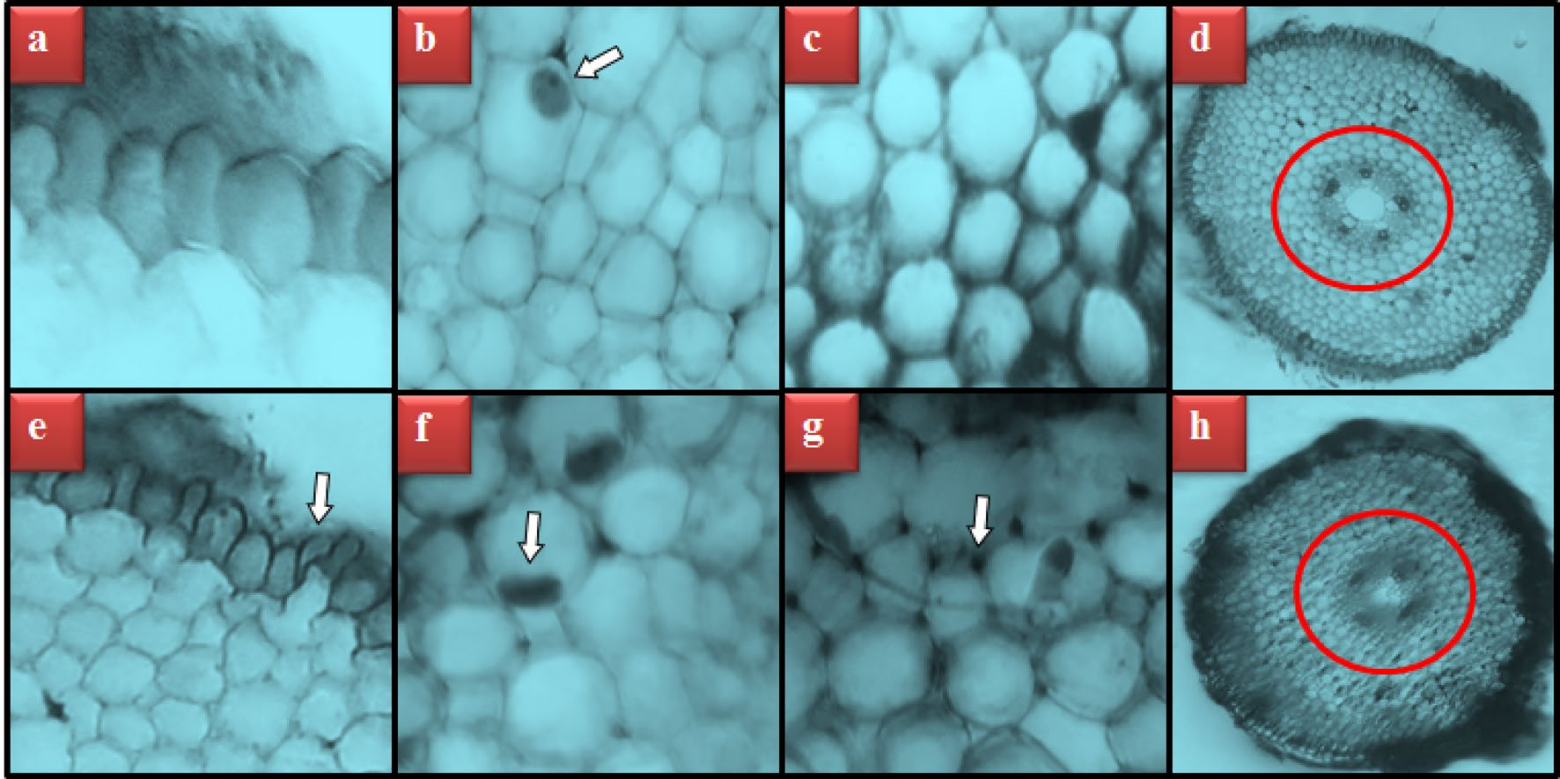
Meristematic cell damages induced by Etoxazole. Epidermis cells with normal appearance (**a**), cell nucleus with normal appearance (*oval*) (**b**), cortex cells with normal appearance (**c**), transmission tissue with normal appearance (**d**), epidermis cell damage (**e**), flattened cell nucleus (**f**), thickening of cortex cell walls (**g**), indistinct transmission tissue (**h**).

Etoxazole treatments induced a variety of meristematic cell damages, including epidermis cell damage, thickening of cortex cell walls, flattened cell nucleus and indistinct transmission tissue in *A. cepa* roots (Table 3, Fig. 5). On the other hand, none of these abnormalities were found in the control group (Fig. 5a–d). The severity of meristematic cell damages in bulbs treated with Etoxazole varied depending on the application dose. All damage types were at a minor level in the LLE group. In the MLE group, the levels of epidermis cell damage (Fig. 5e) and flattened cell nucleus (Fig. 5f) increased to moderate, while thickening of the cortex cell walls (Fig. 5g) and indistinct transmission tissue (Fig. 5h) remained at their levels determined in the LLE group. The severity of all types of meristematic injury increased in the HLE group exposed to the highest dose of Etoxazole. A study on Etoxazole-induced meristematic cell damages has not been found in the literature. However, in toxicity studies focusing on pesticides, disruption of meristematic tissue caused by different pesticides has been previously reported in *A. cepa*^50,57–59^. Deformations in epidermis cells can be considered as a precaution taken by plants to prevent the uptake of pesticides into cells. Cells that encounter pesticides seem to be squeezed, possibly to keep the contaminant out. Thickening of the cortex cell walls can be accepted as a similar defense. On the other hand, the pesticide, which was inevitable to reach the inner parts of the cells, caused deformity in the cell nucleus and defects in the conducting tissue. Membrane disorders, as evidenced by increased MDA levels, probably allowed toxic chemicals to ruin the integrity of tissues^59^. Furthermore, these anatomical changes in the structure of the root meristems may have impeded the transport of water from the environment to the cells, ultimately leading to a limitation in plant growth and germination^60^.

**Table 3:** Severity of meristematic cell damages induced by Etoxazole treatments.

Groups	ECD	FCN	TCCW	ITT
Control	−	−	−	−
LLE	+	+	+	+
MLE	+ +	+ +	+	+
HLE	+ + +	+ + +	+ +	+ +

*Control: Tap water, *LLE* Low level Etoxazole (0.125 mL/L), MLE: Medium level Etoxazole (0.25 mL/L), *HLE* High level Etoxazole (0.5 mL/L). *ECD* epidermis cell damage, *FCN* flattened cell nucleus, *TCCW* thickening of cortex cell walls, *ITT* indistinct transmission tissue. ( −): absent, ( +): minor damage, (+ +): moderate damage, (+ + +): major damage.

Table 4 summarizes the changes in all parameters investigated to clarify the mechanism of dose-dependent toxicity of Etoxazole in onion root meristem cells.

**Table 4:** An overview of how Etoxazole causes toxicity in *A. cepa* cells.

Parameters	LLE	MLE	HLE
Germination percentage	↓	↓↓	↓↓↓
Root elongation	↓	↓↓	↓↓↓
Weight increase	↓	↓↓	↓↓↓
MI score	↓	↓↓	↓↓↓
MN frequency	↑	↑↑	↑↑↑
ACs frequency	↑	↑↑	↑↑↑
MDA level	↑	↑↑	↑↑↑
CAT activity	↑	↑↑↑	↑↑
SOD activity	↑	↑↑↑	↑↑
Meristematic cell damages	↑	↑↑	↑↑↑

*LLE Low level Etoxazole (0.125 mL/L), *MLE* Medium level Etoxazole (0.25 mL/L), *HLE* High level Etoxazole (0.5 mL/L). Downward arrow (↓) symbolizes a decrease and upward arrow (↑) symbolizes an increase in values. The number of arrows indicates the degree of change compared to the control.

## Conclusion

Indiscriminate application of agrochemicals leads to a huge pollution of nature. Due to pesticides accumulating in the environment, many non-target organisms become “toxicity targets”. In this study, the dose-related toxic effects of Etoxazole in *A. cepa*, which is a very popular organism for toxicity studies, were revealed for the first time from a multidimensional perspective. Physiological and genotoxicity parameters showed that Etoxazole suppressed growth, limited cell proliferation and increased the frequencies of MN and ACs. Considering growth arrest and alterations of the levels of genotoxicity indicators, Etoxazole harms the non-target systems in a dose-related way. In addition, study data revealed that Etoxazole is a trigger of oxidative stress as well as a genotoxicity initiator in *A. cepa*. Although the enzymatic antioxidant system was activated following Etoxazole treatments, SOD and CAT activities were significantly reduced due to the highest dose of Etoxazole. Meanwhile, lipid peroxidation continued to elevate due to increasing Etoxazole doses. Furthermore, Etoxazole provoked an apparent deterioration in meristematic cell integrity in roots. All in all, Etoxazole harms non-target systems in a dose-dependent way, considering the results of all the parameters. It should not be forgotten that all biota is at risk and the fate of the environment is in our hands (Supplementary Information). More conscious use of pesticides should be encouraged

## Supplementary Information

Supplementary Information.
