# Supplementary material for: Risk assessment of oxidative stress and multiple toxicity induced by Etoxazole
Source: Sci Rep. 2022 Nov 28;12:20453. doi: 10.1038/s41598-022-24966-0 (PMC9705279; doi:10.1038/s41598-022-24966-0)
Supplement: Supplementary file 1 — Supplementary Information. [file 41598_2022_24966_MOESM1_ESM.docx]

**Supplementary File for Fig. 1.**

The effect of Etoxazole on MI and MI (%).

| **Damage type** | **Control** | **LLE** | **MLE** | **HLE** |
| --- | --- | --- | --- | --- |
| **MI** | 923±20.18^a^ | 856±17.45^b^ | 774±14.53^c^ | 650±11.69^d^ |
| **MI (%)** | (9.23) | (8.56) | (7.74) | (6.50) |

Control: Tap water, LLE: Low level Etoxazole (0.125 mL/L), MLE: Medium level Etoxazole (0.25 mL/L), HLE: High level Etoxazole (0.5 mL/L). Different letters (a-d) indicate the statistical significance between the mean values (p<0.05).

**Supplementary File for Fig. 3.**

Etoxazole toxicity on selected biochemical parameters.

| Groups | **MDA**  **(µM g^-1^ FW)** | **SOD**  **(U mg^-1^ FW)** | **CAT**  **(OD_240 nm_ min.g^-1^ FW)** |
| --- | --- | --- | --- |
| Control | 7.8±0.96^d^ | 32.5±4.16^d^ | 0.64±0.32^d^ |
| LLE | 11.5±1.22^c^ | 37.6±5.35^c^ | 1.45±0.58^c^ |
| MLE | 16.8±1.57^b^ | 51.7±7.50^a^ | 2.52±0.94^a^ |
| HLE | 21.4±1.83^a^ | 44.9±6.72^b^ | 1.98±0.74^b^ |

*Control: Tap water, LLE: Low level Etoxazole (0.125 mL/L), MLE: Medium level Etoxazole (0.25 mL/L), HLE: High level Etoxazole (0.5 mL/L). Different letters (a-d) indicate the statistical significance between the mean values (p<0.05).
